# Enrichment of Omnivorous Cercozoan Nanoflagellates from Coastal Baltic Sea Waters

**DOI:** 10.1371/journal.pone.0024415

**Published:** 2011-09-26

**Authors:** Kasia Piwosz, Jakob Pernthaler

**Affiliations:** 1 Department of Fisheries Oceanography and Marine Ecology, Sea Fisheries Institute in Gdynia, Gdynia, Poland; 2 Limnological Station, Institute of Plant Biology, University of Zurich, Kilchberg, Switzerland; Université Paris Sud, France

## Abstract

Free-living nano-sized flagellates are important bacterivores in aquatic habitats. However, some slightly larger forms can also be omnivorous, i.e., forage upon both bacterial and eukaryotic resources. This hitherto largely ignored feeding mode may have pronounced implications for the interpretation of experiments about protistan bacterivory. We followed the response of an uncultured group of omnivorous cercozoan nanoflagellates from the Novel Clade 2 (Cerc_BAL02) to experimental food web manipulation in samples from the Gulf of Gdańsk (Southern Baltic Sea). Seawater was either prefiltered through 5 µm filters to exclude larger predators of nanoflagellates (F-treatment), or prefiltered and subsequently 1∶10 diluted with sterile seawater (F+D-treatment) to stimulate the growth of both, flagellates and bacteria. Initially, Cerc_BAL02 were rapidly enriched under both conditions. They foraged on both, eukaryotic prey and bacteria, and were highly competitive at low concentrations of food. However, these omnivores were later only successful in the F+D treatment, where they eventually represented almost one fifth of all aplastidic nanoflagellates. By contrast, their numbers stagnated in the F-treatment, possibly due to top-down control by a concomitant bloom of other, unidentified flagellates. In analogy with observations about the enrichment of opportunistically growing bacteria in comparable experimental setups we suggest that the low numbers of omnivorous Cerc_Bal02 flagellates in waters of the Gulf of Gdańsk might also be related to their vulnerability to grazing pressure.

## Introduction

Nanoplanktonic flagellates (NF) are important grazers within aquatic microbial food webs. However, they do not represent a homogenous functional guild of predators: members of the smallest size class (<5 µm) are typically responsible for the major part of picoplankton (bacterial and picocyanobacterial) mortality, while larger species may also forage on algae or other NF [1]. Some NF have been reported to be highly prey-specific and may, e.g., select for or against particular bacterial groups [2], [3], [4], while others seem to have wider food spectra [5], [6].

Some groups of NF may even explore both, bacteria and eukaryotes as a food source [7], possibly with a preference for one type of prey over the other. Such omnivorous species might have multifarious impact on the trophic relationships in aquatic ecosystems. In general, omnivory is hypothesised to affect food web topology, and reduce trophic cascades, as often observed in aquatic environments after removal of top and intermediate grazers [8], [9]. The presence of omnivors may, moreover, modify the response of food webs to perturbations (e.g. eutrophication [10]) by stabilizing the dynamics of such enriched systems [11]. Omnivorous species at an intermediate position in the food web seem to be especially apt to act as intra-guild predators (IG predators) [12]. Intra-guild predation (IGP) is defined as the competition of two species for common resources, and the simultaneous foraging of one of them on its competitor [13]. This phenomenon is well described in terrestrial ecosystems [12], and it can modify both, habitat preference and behaviour of the involved species [14].

In aquatic ecosystems, many ciliated protists have been found capable of feeding on both, bacterial and eukaryotic prey. By contrast, such data are scarce for NF, and the current models and experimental approaches to study aquatic microbial food webs do not consider omnivory within this group of organisms [15]. In theory, omnivorous NF species (i.e. able to feed both on bacterial and eukaryotic cells) could either enhance the total grazing pressure on bacteria, or decrease it by removing other bacterial predators. Moreover, within the framework of the IPG concept omnivorous NF could simultaneously forage upon and compete with other, bacterivorous NF.

The Baltic Sea is a semi-closed basin with narrow and shallow connection to the North Sea. High riverine run-off and reduced water exchange with the oceanic waters results in vertical and horizontal salinity gradients, from 30 PSU in Kattegat to <1 PSU in the northern reaches of the Bothnian Bay. Anthropogenic pressure on the Baltic Sea is very high, and the ecosystem suffers from pollution and eutrophication. Communities of microorganisms present is the Baltic Sea are a mixture of marine, brackish and freshwater species [16], [17], [18], making it a unique site for ecological studies.

We performed food web manipulation experiments to investigate the response to perturbation of a group of omnivorous NF that were also present in surface waters of the coastal Baltic Sea (an uncultured, heterotrophic group of cercozoans from the Novel Clade 2; [19]). We followed the changes in numbers, cell size and food preference (bacteria vs. eukaryotes) of these flagellates upon alteration of prokaryotic community structure, NF intra-guild competition, and the reduction of NF top-down control by larger predators. In addition, we also assessed the population development of an exclusively bacterivorous NF group [7] that was common in the original water sample.

## Results

### Bacteria

**Figure 1:**
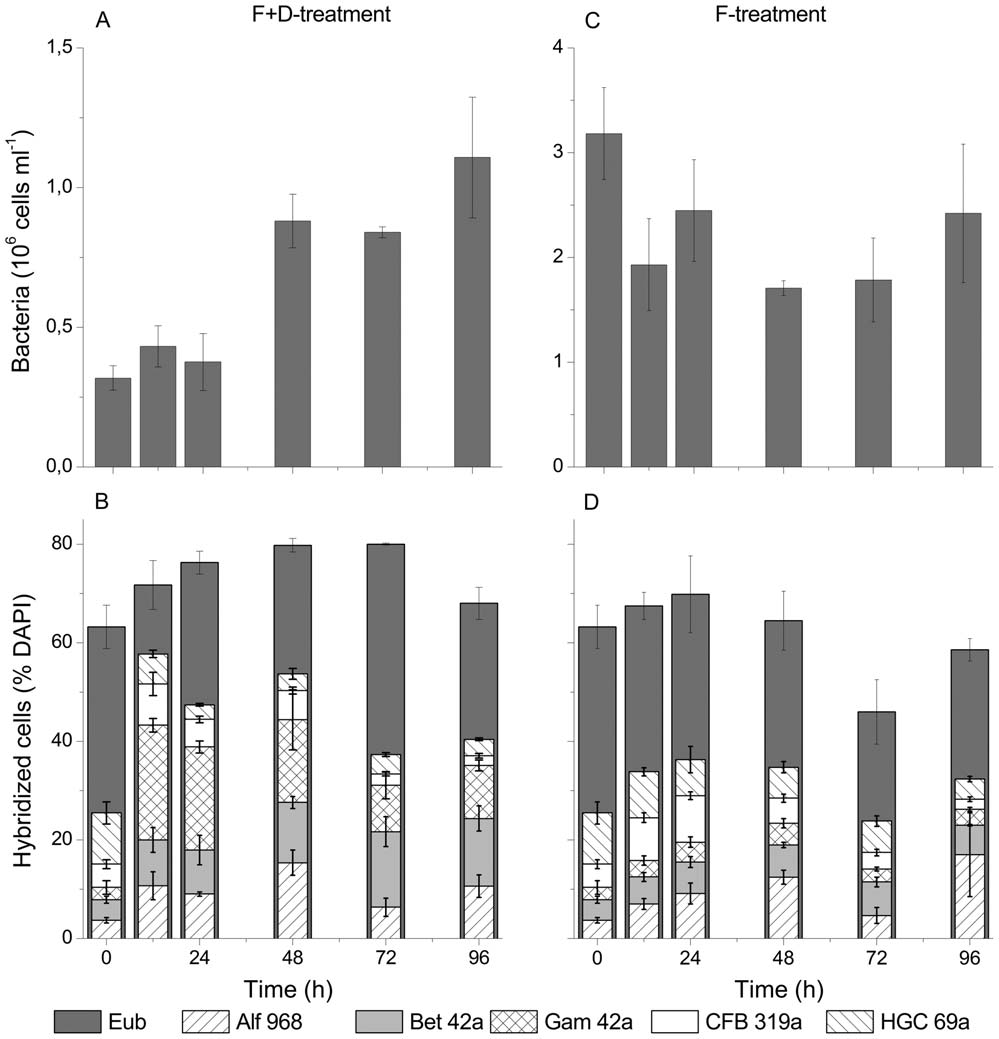
Development of bacterial communities in the experiments. Numbers of bacteria (A & C); and composition of bacterial community (B & D) in the F+D- (5 µm prefiltration and 1∶10 dilution) and F- (5 µm prefiltration) treatments. Error bars show standard deviation, based on the triplicate samples. Eub – bacteria targeted by the general probe Eub I-II-III, Alf968 – Alphaproteobacteria (probe Alf 968), Bet42a – Betaproteobacteria (probe Bet42a), Gam42a – Gammaproteobacteria (probe Gam42a), CFB319a – Cytophaga–Flavobacteria (probe CFB319a), HGC69a – Actinobacteria (probe HGC69a). Note that the Y-axis scale differs between the panels.

The bacterial numbers in the F+D-treatment doubled from t_24_ to t_48_. Before and after this period, the total numbers of bacteria remained relatively constant (Fig. 1A). Hybridization rate with the probe Eub I-II-III ranged from initial 63% to 80% (at t_72_) of total (i.e. DAPI-stained) cells (Fig. 1B). The overall identification rate of the DAPI-stained cells by the sum of all group specific probes varied from only 25% at t_0_ to 58% at t_12_. We observed a substantial increase in the proportions of *Proteobacteria*: initially a 10-fold increase of *Gammaproteobacteria*, followed by a threefold increase in *Alphaproteobacteria*, and later also by *Betaproteobacteria* (Fig. 1B). *Actinobacteria* and members of the *Cytophaga–Flavobacteria* lineage of *Bacteroidetes* (as targeted by probe CFB319a) slightly decreased during the incubation.

The total number of bacterial cells in the F-treatment decreased to approximately half within the first 12 h, and remained more or less constant thereafter (Fig. 1C). Hybridization with probe EubI-II-III detected a maximum of 70% of the DAPI-stained cells, whereas the sum of the group-level probes covered up to 36% of the total DAPI counts (Fig. 1D). Bacterial groups that grew in that treatment were members of the *Cytophaga–Flavobacteria* lineage of *Bacteroidetes* and *Alphaproteobacteria*. *Actinobacteria* decreased, and *Beta-* and *Gammaproteobacteria* remained low during the incubation period.

### Nanoflagellates

**Figure 2:**
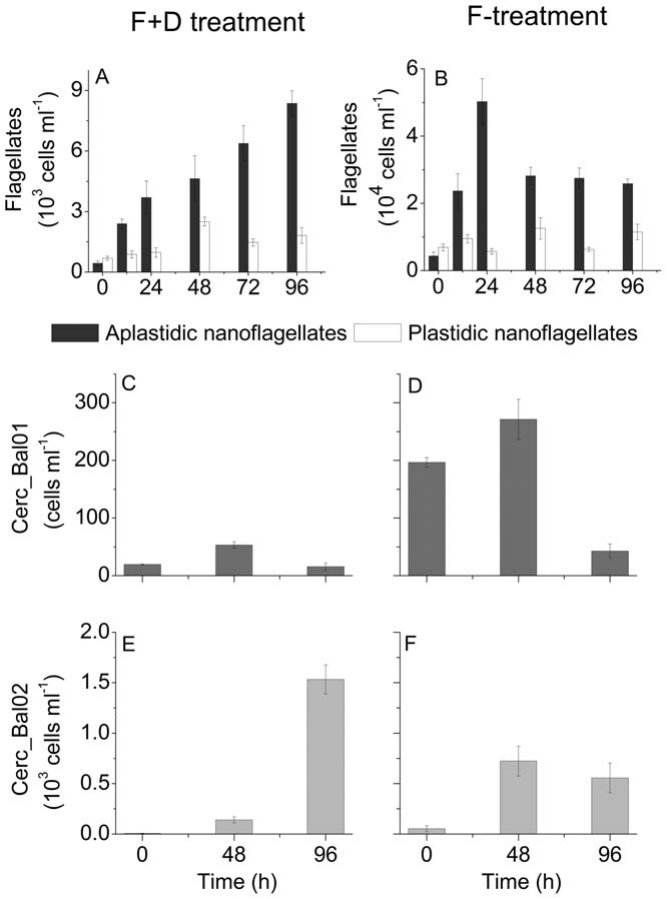
Development of NF in the experiments. Changes of the numbers of plastidic and aplastidic nanoflagellates (A–B), of cercozoan cells targeted by the probe Cerc_Bal01 (C–D); and of cercozoan cells targeted by the probe Cerc_Bal02 (E–F) in the F+D- (5 µm prefiltration and 1∶10 dilution) and F- (5 µm prefiltration) treatments. Error bars show standard deviation, based on the triplicate samples. Note that the Y-axis scale differs between the panels.

Initially, plastidic NF were slightly more numerous than aplastidic ones in both treatments, but were overgrown within 12 h (Fig. 2A, B). The number of aplastidic NF in the F+D-treatment increased almost linearly from 0.4±0.1 to 8.4±0.6×10^3^ cells ml^−1^ (Fig. 2A). In the F-treatment, aplastidic NF grew more than 10-fold during the first 24 h, forming a peak of 5.0±0.7×10^4^ cells ml^−1^. They decreased to 2.8±0.3×10^4^ cells ml^−1^ at t_48_, and varied only slightly thereafter (Fig. 2B).

Changes in the numbers of cells targeted by the probe Cerc_Bal01 were similar in both treatments (Fig. 2C, D). A slight increase during the first 48 h of incubation was followed by a substantial decrease thereafter. The contribution of Cerc_Bal01 cells to the total number of aplastidic NF declined from around 4% to <0.2% in both treatments.

Cells targeted by the newly designed probe Cerc_Bal02 were of minor importance at t_0_ in both treatments (Fig. 2E, F). These cercozoans were also present in environmental samples from April to October 2007, albeit at comparatively low densities (<60 cells ml^−1^, average 16.2±16.7 cells ml^−1^, Fig. S1). In the F+D-treatment only a few Cerc_Bal02 cells were present at t_0_, but they increased to 139±30 cells ml^−1^ at t_48_, yielding an apparent growth rate of 1.7±0.3 d^−1^. This rapid growth moreover continued at an estimated rate of 1.2±0.1 d^−1^, to 1533±144 cells ml^−1^ at t_96_ (Fig. 2E). Cerc_Bal02 grew exponentially through the whole incubation time, giving the apparent growth rate of 1.1±0.1 d^−1^ for this time period and increasing contribution to the total number of aplastidic NF from <1% to 18%. Members of this cercozoan clade initially also grew in the F-treatment (Fig. 2F), at a comparably high apparent growth rate of 1.3±0.3 d^−1^. However, their net growth subsequently ceased, and the numbers of Cerc_Bal02 cells even slightly declined. Their final contribution to the total numbers of aplastidic NF in the F-treatment was only about 2.5%.

**Figure 3:**
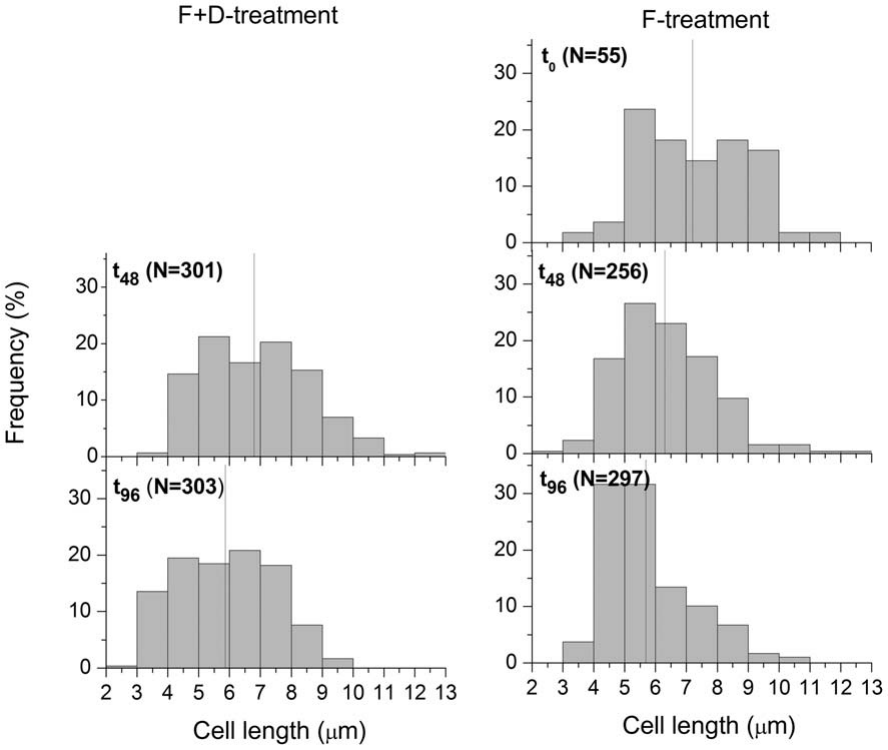
Cell length distribution of the Cerc_Bal02 in the experiments. Changes in the cell length distribution of the cercozoan targeted by the probe Cerc_Bal02 in the F+D- (5 µm prefiltration and 1∶10 dilution) and F- (5 µm prefiltration) treatments. The data from the triplicates were pooled and are shown together on a single graph. Numbers of analysed cells (N) are given in parentheses. The data from the t_0_ point for the 1∶10 dilution treatment are not available because of low numbers of cells that could be found and measured. The black, vertical lines represent the mean cercozoan cell length.

Assuming that the initial distribution of cell-length was similar in both treatments, a shift in size of Cerc_Bal02 cercozoans towards smaller cells in both treatments was observed (Fig. 3). These changes were gradual in the F+D-treatment, where the smallest cells (<6 µm) became more numerous while the proportions of larger individuals did not substantially decline. Size reduction was much more pronounced in the F-treatment. The initially slightly bimodal distribution changed to a unimodal one at t_48_, mainly due to a decrease in the contribution of the largest cells (>9 µm). Cells in the smallest size classes became clearly dominant at the end of the incubation (Fig. 3).

### Food availability and prey selection of Cerc_Bal02

Both treatments likely resulted in elevated grazing pressure on bacteria, as estimated from the decreased ratio of bacteria to total aplastidic NF (Table 1). In the F+D-treatment, high competition of Cerc_Bal02 cells for eukaryotic prey was indicated by their respective ratios at the end of the experiment. Competition for this resource was generally lower in the F-treatment.

**Table 1:** Ratios (mean ± SD) of possible prey-predator pairs on the F+D- and F-treatments at different time points.

	F+D-treatment					
	t_0_	t_12_	t_24_	t_48_	t_72_	t_96_
Bac : aNF	753±122	182±36	103±22	196±37	132±15	133±29
NF : Cerc_Bal02	267±159	—	—	52±10	—	6.6±0.6
Bac : Cerc_Bal02	7.4±3.9×10^4^	—	—	7.6±2.0×10^3^	—	732±194

	F-treatment					
	t_0_	t_12_	t_24_	t_48_	t_72_	t_96_
Bac : aNF	753±122	85±31	50±16	61±7	65±18	93±23
NF : Cerc_Bal02	267±159	—	—	58±12	—	69±17
Bac : Cerc_Bal02	7.4±3.9×10^4^	—	—	2.4±0.4×10^3^	—	4.4±0.9×10^3^

Bac, Bacteria, (a)NF: (aplastidic) nanoflagellates. The uncertainty of these values at t_0_ in the F+D-treatment is relatively higher due to low number of Cerc_Bal02 cells that were counted.

Nanoflagellates were considered bottom-up controlled when the ratio value fell below 1∶100 (based on Gasol [37]).

**Figure 4:**
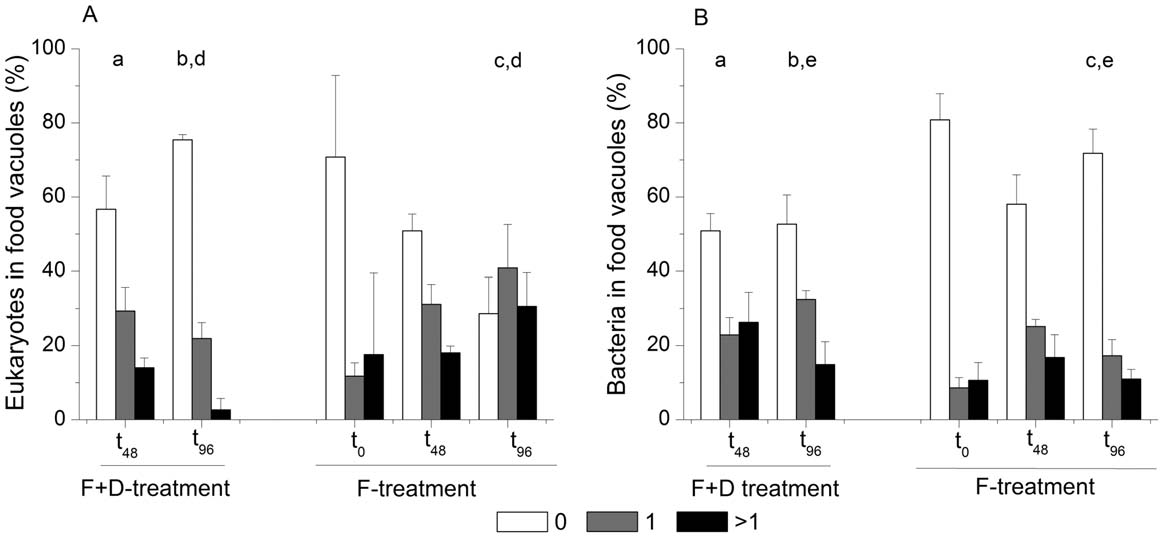
Food preferences of the Cerc_Bal02 in the experiments. Changes in preferred prey of the cercozoan Cerc_Bal02, shown as percentage of the flagellate cells with none, 1 or >1 of eukaryotic or bacterial cells in food vacuoles in (A) the F+D- (5 µm prefiltration and 1∶10 dilution) and (B) F- (5 µm prefiltration) treatments. The data from the t_0_ timepoint for the F+D-treatment are not available because of low numbers of cells that could be found and analysed. Only significantly different timepoints (p<0.05, χ^2^-test) are indicated by the corresponding letters.

We found both prey types (bacteria and eukaryotes) in food vacuoles of cells targeted by probe Cerc_Bal02 (Fig. 4). The experimental treatments caused substantial changes in the ingestion patterns of prey types. At t_48_ the proportion of Cerc_Bal02 cells with no ingested prey items did not vary among treatments. However, significantly higher proportions of cercocoans in the F+D-treatment had ingested bacteria than eukaryotic prey (χ^2^ = 16.05, P<0.001) (Fig. 4). Cerc_Bal02 cells with ingested eukaryotic prey became even less frequent at t_96_ (χ^2^ = 40.87, P<0.0001). In the F-treatment, approximately 80% of cells had empty food vacuoles at t_0,_ which probably reflects the original situation in the field. The proportion of feeding cells was increasing during the incubation, and after 96 h more than 70% of Cerc_Bal02 cells had ingested at least a single eukaryote (Fig. 4). In contrast, bacteria remained significantly less abundant in the food vacuoles of Cerc_Bal02 cells than eukaryotes at that time point (χ^2^ = 112.6, P<0.0001; Fig. 4).

At the end of the experiment the food ingestion patterns of Cerc_Bal02 cells clearly differed between the two treatments. Eukaryotic food items prevailed in the food vacuoles of Cerc_Bal02 cells in the F-treatment (χ^2^ = 151.3, P<0.0001), while ingestion of bacteria was more commonly encountered in the F+D-treatment (χ^2^ = 19.89138, P<0.0001).

**Figure 5:**
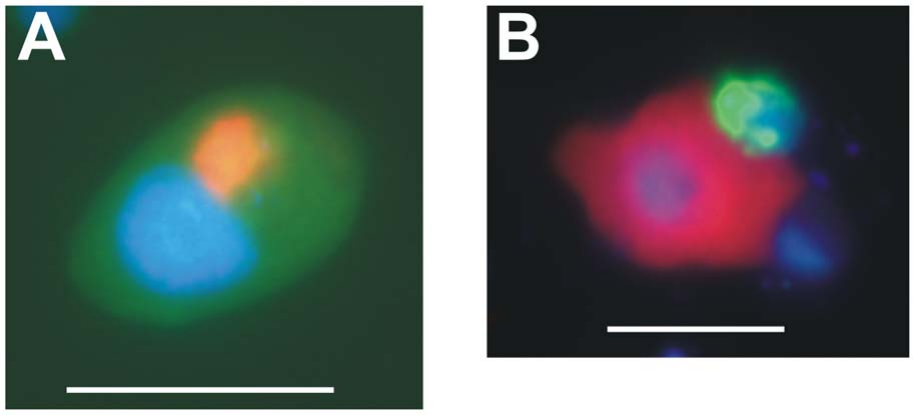
Photomicrographs of hybridized Cerc_Bal02 cells. (A) Photomicrograph of an unknown protist (olive-green) in the experimental enrichments with an ingested cell detected by probe Cerc_Bal02 (red); (B) Cercozoan cell detected with probe Cerc_Bal02 (red) ingesting a cell detected by probe Cerc_Bal01 (green). Blue objects in both panels: DAPI stained nuclei. Scale bars are 10 µm in panel A and 5 µm in panel B. Depictions are true-colour images from the same microscopic fields obtained by simultaneously exciting with several wave lengths.

We also attempted to specifically investigate the influence of Cerc_Bal02 on the numbers of bacterivorous Cerc_Bal01 NF [7] (Fig. 2C, D). However, too few individuals of Cerc_Bal02 with ingested Cerc_Bal01 cells could be observed to reliably quantify the possible impact. Therefore only a qualitative evidence for the existence of this predator-prey relationship was obtained (Fig. 5).

## Discussion

### Experimental food web manipulations

The interactions between flagellate species (e.g. grazing, competition) may influence the composition of the pro- and eukaryotic microbial communities, and hence, the functioning of ecosystems [20]. Therefore, they have been relatively well studied in culture at the species level [21], [22]. However, the results from such laboratory studies cannot be directly transferred even to more complex experimental designs [23], let alone to natural multispecies microbial assemblages. In our study we manipulated whole microbial communities in order to identify NF taxa that are particularly apt to respond to changing growth conditions. We either removed top-down control of the autochthonous NF (prefiltration), or additionally also relieved bacteria from bottom-up limitations (prefiltration followed by dilution), thus altering the competitive context for the success of particular NF taxa.

A fractionation of microbial assemblages through filters with a pore size of 5 µm is typically applied to assess the role of NF in controlling the composition of bacterial communities [2], [24]. Such a treatment is thought to remove the larger predators of flagellates, thereby increasing the numbers of small, allegedly bacterivorous forms and hence, the grazing pressure on bacteria. It has been observed that this manipulation may lead to a shift towards grazing-resistant bacterial taxa, often forming indigestible morphotypes [25], [26], [27]. Such studies typically ignore the possibility that small omnivorous NF might also pass through the filters, profit from the simultaneous absence of top predators and the ample availability of consumable resources (Fig. 2e), and in results relieve top-down control on bacteria [28].

Dilution experiments have been originally introduced as a means of simultaneous estimation of growth and mortality rates of phytoplankton [29] and bacteria [30], bacterial mortality due to viral lysis [31], and for determining conversion factors of leucine and thymidine incorporation for estimating bacterial production [32]. However, the consequences of this treatment appear to be more complex: Dilution appears to selectively favour the most rapidly growing, usually easily cultivable bacterial species by simultaneously increasing the available substrate per microbial cell [33] and reducing the concentrations of protistan predators [34]. Bacteria may be even further relieved from bottom-up limitations by the additional input of DOC and nutrients from phytoplankton cells that are broken during the filtration procedure. In addition, this experimental manipulation may induce a transient resource control on bacterivores by diluting bacteria below the threshold that would sustain NF growth.

We additionally modified the classical dilution treatment by first removing larger protists and metazoans via filtration. This allowed us to directly assess the additional effect of dilution on the studied microbes. For example, there was a clear initial shift of the bacterial assemblage towards *Gammaproteobacteria* in the F+D treatment only. This group has been described to harbour grazing-vulnerable bacteria with an opportunistic growth strategy [34], [35], suggesting that the additional manipulation by dilution relieved bacteria from both, bottom-up and top-down control. However, a pronounced bacterial bloom, as observed in other dilution experiments [36], did not occur in our experiments, suggesting that grazing pressure by NF was quickly re-established. In view of the initially rapid growth of the bacterivorous clade Cerc_Bal01, its subsequent reduction and the simultaneous increase of the omnivorous Cerc_Bal02 cercozoans (Fig. 2C, E), we concluded that bacteria in the F+D treatment may have been first controlled by bacterivores only, and subsequently mainly by the omnivores, including the investigated cercozoan groups.

In theory, there ought to be different mechanisms of NF control between the treatments, namely in the F+D-treatment expected bloom of bacteria would later relieve NF from the bottom-up control [36], while in the F-treatment competition for resources was predicted to be strong [2]. We assessed the possible control mode on NF by calculating prey (bacteria) to predator (NF) ratios (Table 1). The boundary value of this ratio for bottom-up vs. top-down control of NF was estimated to be about 100∶1, based on the equation for mean realized abundance given by Gasol [37]. In agreement with theoretical predictions, bottom-up control on NF (and hence top-down on bacteria) was higher in the F-treatment. The difference between the treatments was statistically significant (Mann-Whitney U-test, Z = 4.10, p<0.0001) but not substantial (Table 1). In contrast, eukaryotic prey was less available for the Cerc_Bal02 cells in the F+D-treatment (Mann-Whitney U-test, Z = −2.16, p = 0.03), which could have promoted the observed relatively high ingestion of bacteria. Considering that the proportion of the Cerc_Bal02 cells with empty food vacuoles in the F+D-treatment was higher (Fig. 4), it can be concluded that, in contrast to theoretical assumptions, these cercozoans were exposed to higher competition in this treatment. Nevertheless, it did not negatively influence their growth, suggesting high competitive ability of this group.

### Highly successful omnivorous cercozoans, possibly top-down controlled

The continuous exponential growth and increased contribution of the omnivorous Cerc_Bal02 at low prey to predator ratios in the F+D-treatment (Table 1) indicated that this flagellate was neither top-down nor bottom-up controlled, and thus it may be competitive both, for eukaryotic and bacterial prey. The competition for resources could have been additionally reduced by directly foraging on its possible bacterivorous competitors (Fig. 5B), which would additionally increase proportion of the Cerc_Bal02 in the NF community. The prey to predator ratios were generally decreasing (Table 1), while the proportion of feeding Cerc_Bal02 cells remained stable (Fig. 4A). Therefore, it seems most plausible that the apparent success of omnivorous Cerc_Bal02 was caused by out-competing other NF. This success of an omnivore puts into perspective previous findings from enrichment cultures suggesting that omnivorous NF are opportunistic species that only grow at high prey concentration, as e.g. observed for *Paraphysomonas imperforata* [38], [39]. In contrast, flagellates affiliated with Cerc_Bal02 may be opportunistic omnivores successful at low food availability.

The observed success of the Cerc_Bal02 population at low prey concentration raises the question about the possible reasons for their low numbers in the studied environment (Fig. S1). The results of the F-treatment point at the importance of top-down control: A removal of larger grazers allowed for an equally rapid growth of the Cerc_Bal02 cercozoans in both treatments (Fig. 2C, D). However, their growth did not continue beyond t_48_ in the F-treatment, although these flagellates still were actively feeding on other NF (Fig. 4). The reduction of the NF bloom in this treatment after only 24 h of incubation, therefore, would suggest that Cerc_Bal02 were mainly controlled by mortality, most likely by the grazing of small predators (Fig. 5A) [40]. Alternatively, this decline of NF numbers might have also been related to viral lysis [41]. In the latter case, however, a similar decrease would have also been expected in the F+D-treatment. Therefore, it is more likely that a large proportion of flagellates in the later phase of the F-treatment might in fact have been omnivores that were foraging on each other, inducing a top-down control also on Cerc_Bal02. Altogether, it might be concluded that Cerc_Bal02 cercozoans may be rare under natural conditions despite being highly competitive at low prey concentration due to their vulnerability to various mortality sources (including grazing by other protists, Fig. 5A).

The presumably distinct grazing pressure on Cerc_Bal02 cells between the treatments might have also contributed to the observed differences in their size distributions (Fig. 3): Assuming the likelihood of being consumed to be proportional to cell age, high predation pressure should lead to an increased formation of small cells, as observed in the F-treatment (Fig. 3). In contrast, Cerc_Bal02 cells of various sizes were almost equally abundant in the F+D-treatment, where no indication for top-down control of Cerc_Bal02 was apparent (as judged from their continuous rapid growth, Fig. 2E).

It should be noted that the exposure to different regimes of grazing pressure and food availability might have also caused the rise of different genotypes within the diverse group of cercozoans targeted by the probe Cerc_Bal02 (Fig. S2). Thus, the observed changes in food preferences and cell size between the treatments might not necessarily reflect the adaptation of a single population only, but could also indicate the success of different ecotypes at different environmental conditions [42]. In either case, our interpretation of the basic ecological factors influencing the success of this group of omnivorous cercozoa in the environment would be still valid.

In summary, our experimental food web manipulation created different scenarios of bottom-up vs. top-down stress for Cerc_Bal02 cercozoans, resulting in contrasting patterns of growth and grazing behaviour. These flagellates were found to be omnivores capable of successful reproduction at low food availability, but seemed to be vulnerable to predation by other NF. This suggests that Cerc_Bal02 cercozoans follow an ‘opportunistic’ life strategy and are possibly controlled by grazers in the environment. Further studies, e.g. of the *in situ* food vacuole content of their potential predators (e.g. ciliates and dinoflagelletes) would be required to confirm this hypothesis.

## Materials and Methods

### Experimental design

Coastal surface water (approx. 400 m off-shore) for the experiments was collected with a clean bucket from the Gulf of Gdańsk (Baltic Sea) on August 20, 2007, prefiltered through a 10 µm plankton net and transported to the laboratory within 15 minutes. Temperature was measured *in situ* with a thermometer, and salinity was determined in the laboratory with an InoLab probe (WTW).

The collected water was further prefiltered through a 5 µm membrane filter (diameter 47 mm, Isopore, Millipore, USA) at low pressure (0.26 bar). Part of the so pre-treated water was directly used for the experiment (F-treatment), while the rest was ten-fold diluted with sterile seawater (filtration through 5, 1.2 and finally 0.22 µm membrane filters, diameter 47 mm, Isopore, Millipore, USA) (F+D-treatment). 2 litres of the pre-treated water were incubated in triplicates in 5 L glass Erlenmayer flasks in the dark at in situ temperature (19.6°C) and salinity (7.2 PSU) for 96 h in SANYO incubators.

### Numbers of bacteria and nanoflagellates

Samples for total numbers of heterotrophic bacteria and nanoflagellates (NF) were taken after 12, 24 h and every 24 h thereafter for a total of 96 h. Bacteria (4–50 ml of water) were fixed with buffered paraformaldehyde solution (pH 7.6, final conc. 1%), and NF (15–100 ml) with alkaline Lugol's solution followed by addition of formaldehyde solution (final conc. 2%) and decolorization with 3% sodium thiosulphate [43]. The fixed samples were filtered onto polycarbonate membrane filters (25 mm diameter, Isopore, Millipore, pore size 0.22 µm for bacteria and 0.8 µm for nanoflagellates), stained with 4′,6-Diamidino-2-phenylindole dihydrochloride (DAPI) (Sigma-Aldrich, Germany) solution (conc. 1 µg ml^−1^ for bacteria and 5 µg ml^−1^ for nanoflagellates) and examined by fluorescence microscopy at UV/Blue excitation/emission wavelengths under 1000× magnification [44]. The presence of chloroplasts in NF cells was determined at green/red excitation/emission.

### Fluorescence In Situ Hybridization and Catalysed Reporter Deposition (CARD-FISH)

#### Bacteria

Samples for CARD-FISH analysis of bacterial groups were collected together with those for the total counts but the tripled volume was filtered on white polycarbonate filters (47 mm diameter, Isopore, Millipore, pore size 0.22 µm). After enzymatic digestion with lysozyme (10 mg ml^−1^, 1 h) and proteinase K (75 nl ml^−1^, 30 mins), bacterial cells were hybridized with horseradish peroxidase labelled oligonucleotide probes [45]. We used the general bacterial probe Eub I-II-III [46], and group specific probes for *Alpha-* (Alf968; [47]), *Beta-* (Bet42a) and *Gammaproteobacteria* (Gam42a; [48]); members of the *Cytophaga–Flavobacteria* lineage of *Bacteroidetes* (CFB319a; [49]), and *Actinobacteria* (HGC69a; [50]). Fluorescence signals were amplified with tyramides (Sigma) labelled by carboxyfluorescein (MolecularProbes, Invitrogen). The evaluation of the preparations was performed by a semi-automatic procedure based on motorized epifluorescence microscopy and image analysis [51], [52]. However, due to the patchiness of the filters, some microphotographs were acquired and evaluated manually.

#### Nanoflagellates

Samples for the determination of the abundance of NF affiliated with two groups of Cercozoa were collected three times: at t_0_, t_48_ and t_96_. They were fixed and filtered as described for the total counts. The adjustment of hybridization condition and staining procedure for the flagellates by CARD-FISH is described in Piwosz and Pernthaler [7]. We used two probes: Cerc_Bal01 [7], and a newly designed probe Cerc_Bal02, a mixture of two oligonucleotides together targeting members of the Clade 2 of the so-called Novel Cercozoan Group [19] (Fig. S2) (Cerc_Bal02A: 5′ – AGA ACC CGT AGT CCT ATA – 3′ and Cerc_Bal02B: 5′ – TTC GAC GTA TAA GGG TGC – 3′; hybridization with 30% formamide). Double hybridizations were performed following the general CARD-FISH protocol but with (i) an additional quenching step of the probe-delivered peroxidases after the first signal amplification and (ii) using tyramides labelled with Alexa_488_ (for the Cerc_Bal01 probe) and Alexa_665_ (for the probe Cerc_Bal02). At least 200 hybridized flagellates per sample were counted in a minimum of 20 microscopic fields by epifluorescence microscopy (AxioImager.M1, Carl Zeiss, Germany) at blue/UV excitation. If the densities of target cells were too low, the complete filter piece was screened (>170 microscopic fields). Apparent growth rates were calculated assuming exponential growth kinetics.

### Size measurements of Cerc_Bal02 cells

Approx. 100 cells from each triplicate hybridized with the Cerc_Bal02 probe were photographed after visualisation by epifluorescence microscopy (AxioImager.Z1, Carl Zeiss, Jena, Germany, 640× magnification) with an AxioCam MR3 camera (Carl Zeiss), and their cell size was determined using the length tool of the AxioVision software (Carl Zeiss).

### Feeding preferences of Cerc_Bal02 cells

The presence of bacteria and eukaryotic prey in food vacuoles was assessed by epifluorescence microscopy based on their DAPI staining (AxioImager.M1, Carl Zeiss) at blue/UV excitation. Prey items were counted only (i) if they were inside a food vacuole, visible as a dark area within a hybridized flagellate cell (Fig. S3 A–D), and (ii) if they were in the same focal plane as the flagellate cell (Fig. S3 E–F), to exclude objects that had settled onto or below the surface of the examined cell [7].

### Statistical analysis

For the analysis of differences in frequency of ingested prey items, a χ^2^ test was performed. To fulfil the assumption of the χ^2^ test of expected value for each group to be >10, the cells were divided into three groups: i) no foot items, ii) a single food item, and iii) >1 food items inside food vacuoles. Differences in the control mode (bottom-up vs. top-down) between the treatments were analysed by the Mann-Whitney U-test.

## Supporting Information

Figure S1 **Numbers of Cerc_Bal02 in the environment.** Abundance of cells hybridized with the probe Cerc_Bal02 in the Gulf of Gdańsk (Southern Baltic) from April to October 2007.
(TIF)

Figure S2 **Target clades for the probes used in the study.** Maximum likelihood (ML) tree of almost complete 18S rDNA sequences of cercozoans showing groups targeted by the probes used in this study. Bootstrap values only >50% (100 ML trees) are depicted, nodes with bootstrap values <50% are collapsed into multifurcations. The naming of the collapsed groups (black trapeziums) follows the notations Cavalier-Smith and Chao [53]. Modified from [7].
(TIF)

Figure S3 **Photomicrographs of hybridized Cerc_Bal02 cells with ingested eukaryotic and bacterial prey inside food vacuoles.** (A) Hybridized cercozoan cell detected with probe Cerc_Bal02 visualized in blue light. A large food vacuole is clearly visible (arrow head); (B) The same cell visualized in UV light (DAPI-staining). A eukaryotic cell inside the food vacuole is indicated by the arrow head. (C) Cerc_Bal02 cell visualized in blue light with two small food vacuoles (arrows) (D) The same cell visualized in UV light. Bacterial cells can be seen inside the food vacuoles (arrows) (E) Cerc_Bal02 cell visualized in blue light without a food vacuole (F) The same cell visualized in UV light. Arrow indicates bacterial cells. Due to lack of visible food vacuoles and different focus plane of bacterial and flagellate cell, this Cerc_Bal 02 cell was classified to contain no prey items.
(TIF)
