# Supplementary figures and images for: Enrichment of Omnivorous Cercozoan Nanoflagellates from Coastal Baltic Sea Waters
Source: PLoS One. 2011 Sep 26;6(9):e24415. doi: 10.1371/journal.pone.0024415 (PMC3180281; doi:10.1371/journal.pone.0024415)

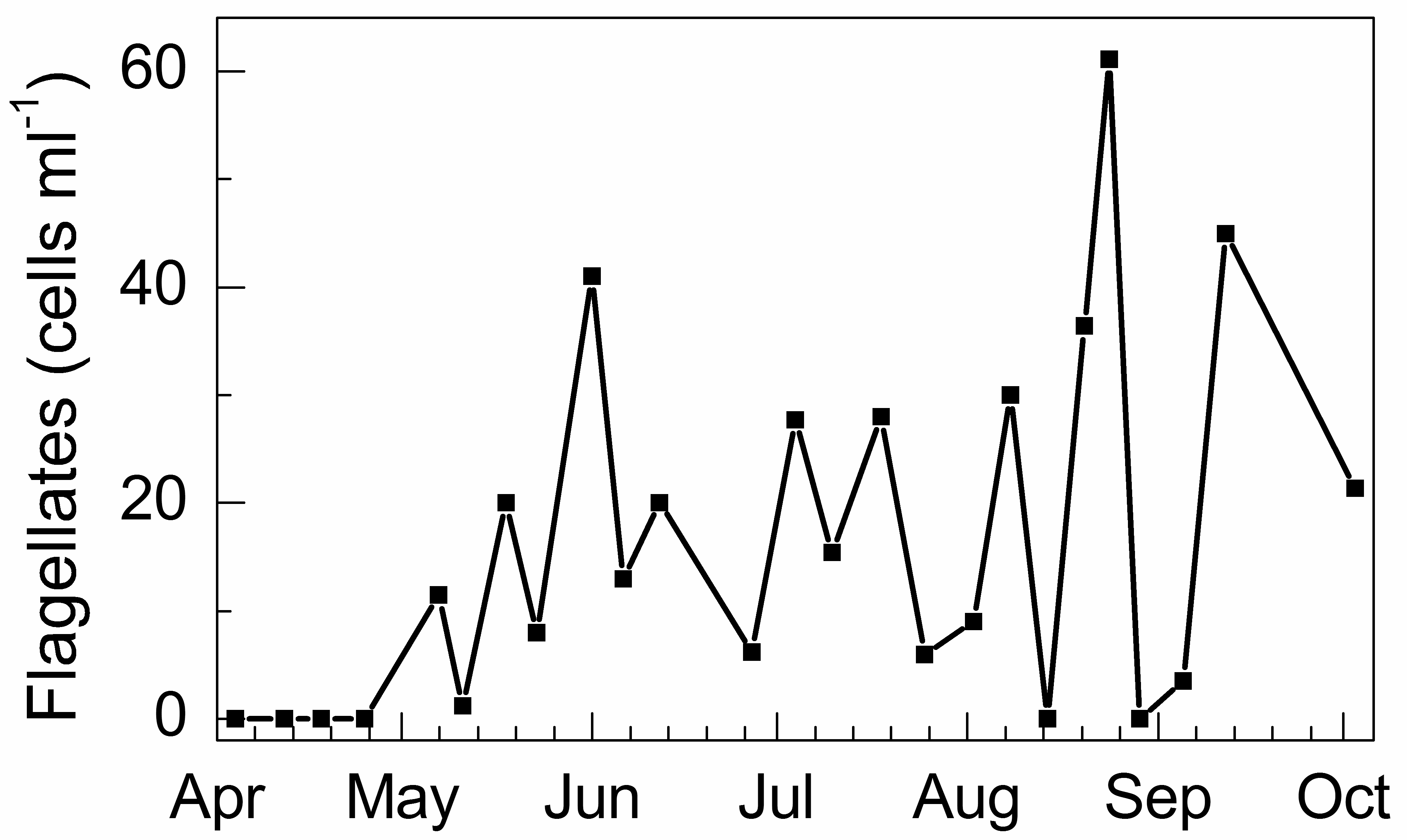

Supplement: Figure S1 — Numbers of Cerc_Bal02 in the environment. Abundance of cells hybridized with the probe Cerc_Bal02 in the Gulf of Gdańsk (Southern Baltic) from April to October 2007. (TIF) [file pone.0024415.s001.tif]

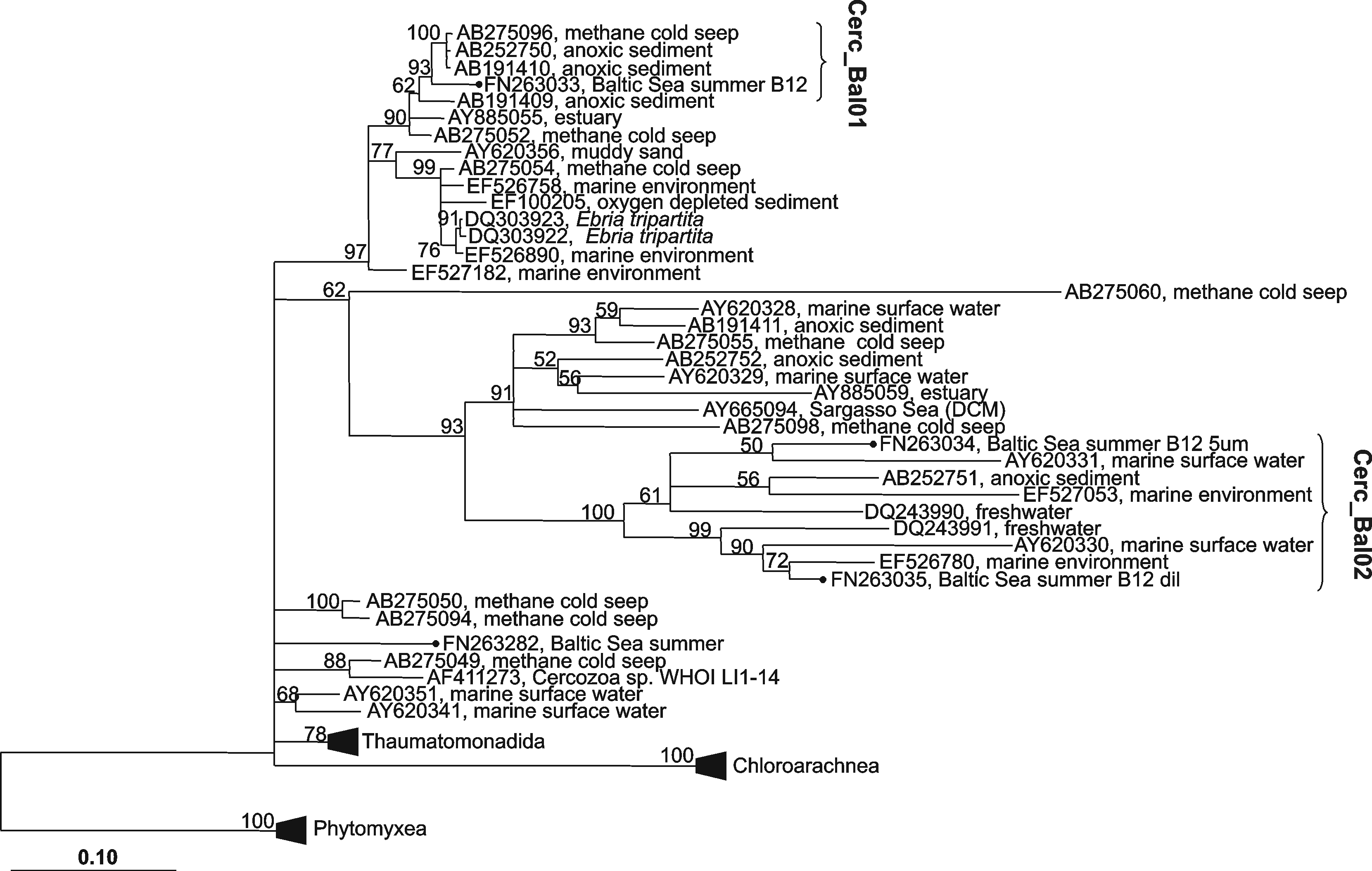

Supplement: Figure S2 — Target clades for the probes used in the study. Maximum likelihood (ML) tree of almost complete 18S rDNA sequences of cercozoans showing groups targeted by the probes used in this study. Bootstrap values only >50% (100 ML trees) are depicted, nodes with bootstrap values <50% are collapsed into multifurcations. The naming of the collapsed groups (black trapeziums) follows the notations Cavalier-Smith and Chao [53]. Modified from [7]. (TIF) [file pone.0024415.s002.tif]

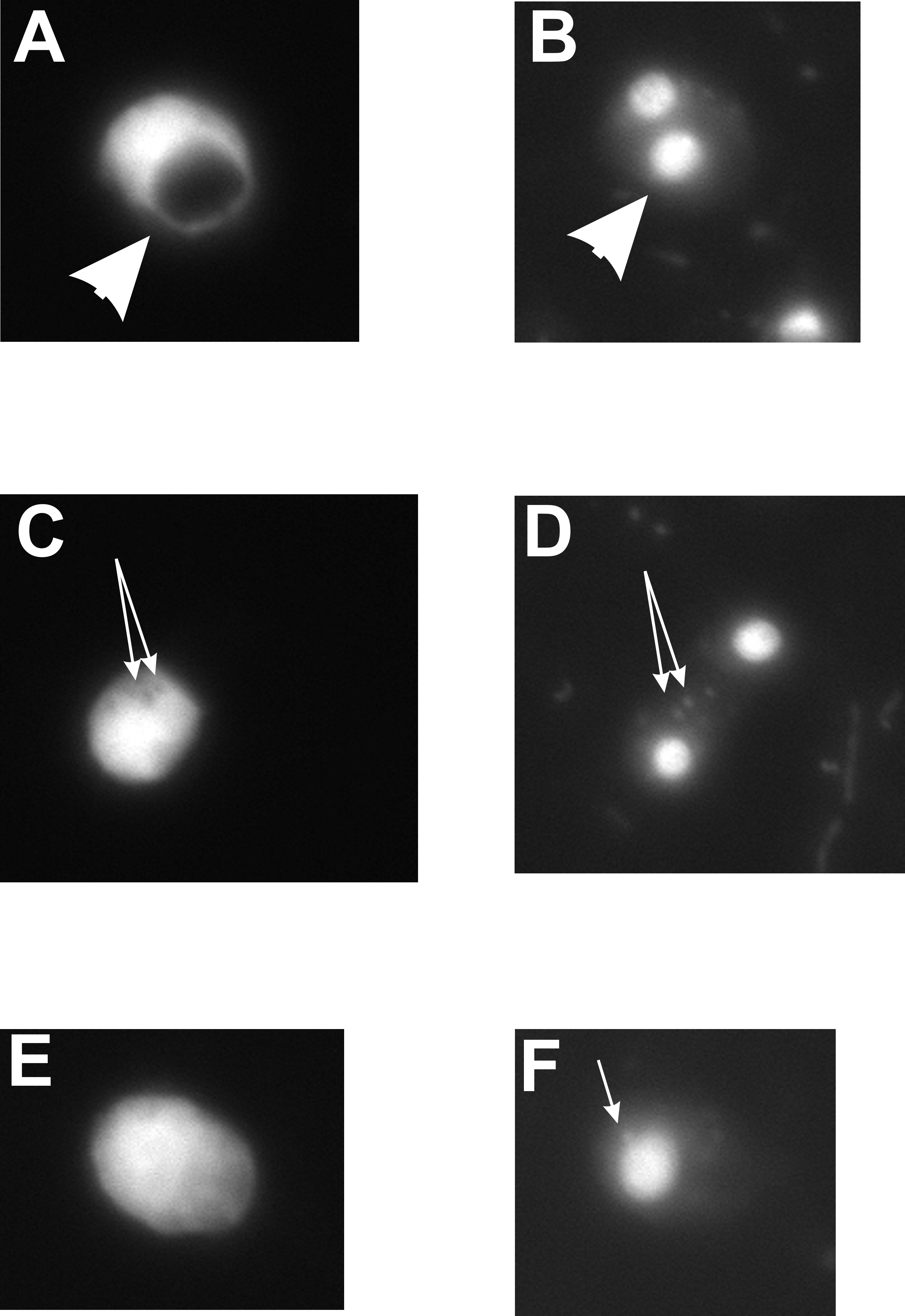

Supplement: Figure S3 — Photomicrographs of hybridized Cerc_Bal02 cells with ingested eukaryotic and bacterial prey inside food vacuoles. (A) Hybridized cercozoan cell detected with probe Cerc_Bal02 visualized in blue light. A large food vacuole is clearly visible (arrow head); (B) The same cell visualized in UV light (DAPI-staining). A eukaryotic cell inside the food vacuole is indicated by the arrow head. (C) Cerc_Bal02 cell visualized in blue light with two small food vacuoles (arrows) (D) The same cell visualized in UV light. Bacterial cells can be seen inside the food vacuoles (arrows) (E) Cerc_Bal02 cell visualized in blue light without a food vacuole (F) The same cell visualized in UV light. Arrow indicates bacterial cells. Due to lack of visible food vacuoles and different focus plane of bacterial and flagellate cell, this Cerc_Bal 02 cell was classified to contain no prey items. (TIF) [file pone.0024415.s003.tif]
